# Effect of Ultrasonic-Assisted Extraction on the Structural and Physiological Activity of Jackfruit Polysaccharides

**DOI:** 10.3390/foods15010132

**Published:** 2026-01-02

**Authors:** Jinmei Hu, Zongcheng Luo, Fengzhen You, Donghui Luo, Fengchuan Ma, Zhongsheng Tang, Siming Zhu

**Affiliations:** 1 College of Food Science and Engineering, Guangdong Ocean University, Yangjiang 529500, China; hjm667788@163.com (J.H.); zongchengluo@foxmail.com (Z.L.); yfz20010620@163.com (F.Y.); luodonghui@gdou.edu.cn (D.L.); mafc1969@163.com (F.M.); 2 Chaozhou Branch of Chemistry and Chemical Engineering Guangdong Laboratory, Chaozhou 521011, China; 3 Yangjiang Research Institute of Guangdong Ocean University, Yangjiang 529500, China; 4 School of Food Science and Engineering, South China University of Technology, Guangzhou 521011, China

**Keywords:** ultrasonic assisted extraction, jackfruit polysaccharide, structural characterization, biological activity, intestinal flora regulation

## Abstract

This study aimed to investigate the effects of ultrasound-assisted extraction (UAE) on the physicochemical properties, biological activities, and intestinal flora regulatory capacity of jackfruit polysaccharides (JPs). Under optimized UAE conditions (liquid-to-solid ratio 30 mL/g, extraction time 30 min, power 90 W), the yield of JP reached 8.70 ± 0.11%. Compared with hot-water-extracted jackfruit polysaccharides (HAE-JPs), ultrasonic-assisted extracted jackfruit polysaccharides (UAE-JPs) exhibited a lower molecular weight, a smaller particle size, and a significant 11.5-fold increase in galacturonic acid content. Structural analyses confirmed that UAE-JPs retained a triple-helix and highly branched conformation but with enhanced exposure of acidic monosaccharides. These structural modifications contributed to superior antioxidant activity and enzyme inhibition ability, demonstrated by its lower IC_50_ values against DPPH, ABTS radicals, and α-glucosidase. Crucially, in vitro fecal fermentation revealed that UAE-JPs and HAE-JPs differentially modulated the gut microbiota. UAE-JPs preferentially promoted the proliferation of *Lactobacillus* (an increase of 27.04%) and *Bifidobacterium*, facilitating short-term acidification. In contrast, HAE-JPs enriched butyrate-producing bacteria like *Clostridium* (increase of 18.56%). Both polysaccharides significantly inhibited the growth of *Fusobacterium* (a decrease of 5.23%) related to cancer. Consequently, this study establishes UAE as a green and efficient technique capable of not only modifying the structure of JPs but also precisely tailoring their prebiotic functionality, which ultimately demonstrates the potential of UAE-JPs as a functional food ingredient with enhanced bioactivity.

## 1. Introduction

The jackfruit (*Artocarpus heterophyllus* Lam.) is an important economic crop in tropical regions, rich in nutrients such as polysaccharides, proteins, fats, and minerals. Studies have shown that the polysaccharides in the flesh of jackfruit have various biological activities, including antioxidant, immune regulation, hypoglycemic, and improvement of intestinal health, etc. [1]. Currently, the most commonly used method for extracting polysaccharides from jackfruit flesh is the traditional hot water extraction method (HAE), which has problems such as low extraction efficiency and harsh conditions, which may lead to the degradation of polysaccharides and affect their structure and activity [2]. Therefore, developing efficient and mild new extraction technologies is of great significance for fully exploring the application potential of jackfruit polysaccharides (JPs).

The extraction process of polysaccharides directly affects their yield, structural characteristics, and functional activity [3]. Ultrasound-assisted extraction (UAE), as a new type of physical field enhancement technology, can effectively damage the cell wall structure through the instantaneous high pressure and microjet generated by the cavitation effect, significantly improving the extraction efficiency [4]. Compared with HAE, UAE has the advantages of low energy consumption, short time, and mild conditions, which help to maintain the natural conformation and biological activity of polysaccharides [5]. This technology has been successfully applied in the extraction of various plant polysaccharides, such as kelp [6], dragon fruit peel [7], and goji berries [8]. Studies have shown that UAE not only increases the yield of polysaccharides but also enhances their functional activity by regulating structural parameters such as molecular weight and monosaccharide composition. For example, the kelp polysaccharides treated by UAE exhibit stronger antioxidant and α-amylase inhibitory abilities, and the probiotic activity of dragon fruit peel polysaccharides is significantly enhanced. Although UAE technology is widely used in the extraction and modification of plant polysaccharides, research specifically targeting JP remains relatively limited. Existing studies have primarily focused on optimizing traditional extraction processes or comparing the basic biological activities of polysaccharides obtained by conventional methods. For example, Zhu et al. [9] employed response surface methodology to optimize the extraction of JPs, achieving a polysaccharide yield of 6.18% under conditions of a solid-to-liquid ratio of 30:1, an extraction time of 2.5 h, and an extraction temperature of 90 °C. In another study, Yu et al. [10] compared the structural characteristics and antioxidant activities of fermented and unfermented JPs extracted using traditional methods. However, systematic investigations into the regulatory effects of UAE on the structure of JPs and the resulting structure–activity relationships underlying its physiological functions are still lacking.

In addition, the gut microbiota, referred to as the “second genome” of the human body, maintains a close relationship with host health through its homeostasis. As an important component of dietary fiber, polysaccharides can be selectively utilized by gut microorganisms, modulating the structure of the microbiota, producing beneficial metabolites such as short-chain fatty acids, and thereby exerting prebiotic effects [11]. Although preliminary studies have suggested that jackfruit polysaccharides may possess the potential to regulate gut microorganisms [12], the specific regulatory effects and mechanisms of ultrasound-assisted extracted jackfruit polysaccharides (UAE-JPs) on the composition and function of the gut microbiota remain to be thoroughly elucidated. Based on this, this study systematically compared the differences in yield, chemical composition, and structural characteristics of jackfruit polysaccharides extracted by UAE and HAE. Furthermore, by integrating an in vitro fecal fermentation model, the regulatory effects of the two types of polysaccharides on gut microbiota were analyzed, aiming to elucidate the intrinsic mechanism by which UAE modulates the prebiotic activity of polysaccharides through structural modification. The findings of this study not only provide a new strategy for the high-value utilization of jackfruit resources but also offer a theoretical basis for the functional modulation of polysaccharides by ultrasonic fields.

## 2. Materials and Methods

### 2.1. Materials

Fresh yellow-fleshed jackfruit was purchased from JD.com, Inc. (Hangzhou, Zhejiang, China). The glucose (≥98%), p-Nitrophenyl-β-D-glucoside (p-NPG), α-glucosylase, α-amylase, Coomassie Brilliant Blue G-250, m-hydroxybiphenyls, and galacturonic acid were purchased from Yuanye Biotechnology Co., Ltd. (Shanghai, China). Salicylic acid, 2,2′-azino-bis(3-ethylbenzothiazoline-6-sulfonic acid) (ABTS), 2,2-diphenyl-1-picrylhydrazyl (DPPH), soluble starch, potassium persulfate, and bovine serum albumin were purchased from Macklin Co., Ltd. (Shanghai, China). All other chemicals were of analytical grade and obtained from Sigma-Aldrich Co., Ltd. (Shanghai, China).

### 2.2. Preparation of Crude Polysaccharides

#### 2.2.1. Pretreatment of Jackfruit Powder

After removing the peel, fruit shreds, and seeds, the flesh of the jackfruit was dried at 60 °C and crushed into jackfruit powder (40-mesh). The jackfruit powder was dispersed in petroleum ether at 60 °C for 5 h to obtain the defatted jackfruit powder and was stored at 4 °C before use.

#### 2.2.2. Extraction Procedure

Ultrasonic-assisted extraction (UAE) is widely used due to its comprehensive advantages over traditional hot water extraction (HAE), alkaline extraction (AE), and microwave-assisted extraction (MAE). The core mechanism lies in the cavitation effect, mechanical vibration, and shear force generated by ultrasound, which can efficiently disrupt the cell wall structure and promote the release of polysaccharides, achieving efficient extraction under mild conditions [5]. Compared with HAE, UAE significantly shortens the extraction time and avoids the degradation of polysaccharides caused by prolonged high temperature; compared with AE, UAE uses only water as the solvent, avoiding the use of strong alkaline reagents and their subsequent neutralization steps, making it more environmentally friendly; compared with MAE, UAE has lower equipment costs and more uniform heat effects, which can better protect the structural integrity of polysaccharides and help maintain their biological activity. Therefore, the UAE, with its efficient, mild, economical, and environmentally friendly characteristics, provides an ideal solution for this study to obtain high-quality polysaccharides.

The crude polysaccharides from the pulp of jackfruit were extracted by the ultrasonic-assisted method, with the traditional hot extraction method as the control. The specific process was as follows: the defatted pulp powder was treated by ultrasonication (30 min, 90 W), and then extracted by magnetic stirring at a solid–liquid ratio of 1:20 g/mL and 60 °C for 60 min. The subsequent steps included centrifugation and filtration, concentration, alcohol precipitation, overnight standing, and then the precipitate was collected by centrifugation and vacuum freeze-dried to obtain the crude polysaccharides. In the single-factor experiments, the effects of solid–liquid ratio (1:15–1:35 g/mL), extraction time (30–150 min), extraction temperature (40–80 °C), ultrasonic time (10–50 min), and ultrasonic power (30–150 W) on the extraction effect were investigated under the condition of fixing other factors [13].

#### 2.2.3. Response Surface Optimization (RSM)

Based on the single-factor experiment (Section 2.2.2), the solid–liquid ratio (A), ultrasonic time (B), and ultrasonic power (C) were selected as independent variables, and the yield of polysaccharides was taken as the response value for RSM (Table S1). A 3-factor, 3-level Box–Behnken response surface center combination test design was conducted.

### 2.3. Purification of Crude Polysaccharides in Jackfruit Flesh

The deproteinization and decolorization treatments were performed by the enzymatic method combined with the TCA method and macroporous resin adsorption [14,15]. The resulting polysaccharide solution was dialyzed (3000 Da for 72 h) and freeze-dried. The freeze-dried powder was preliminarily purified by DEAE-Cellulose 52 column chromatography [16]. The fraction with the highest polysaccharide content was collected and further purified by Sephadex G-100 gel column to obtain refined jackfruit polysaccharide (JP). JPs prepared by optimized ultrasonic-assisted heating extraction conditions (solid–liquid ratio 30 g/mL, ultrasonic time 30 min, ultrasonic power 90 W, extraction time 60 min, extraction temperature 60 °C) and heating extraction (60 min, 60 °C) were named UAE-JPs and HAE-JPs, respectively.

### 2.4. Structural Characterization Methods

The protein content was measured by the Coomassie Brilliant Blue method [17]. The uronic acid content was determined using the m-hydroxydiphenyl method [18]. Monosaccharide composition was analyzed according to the report [19].

The UV–Vis spectrum of JP was tested by a TU-1901 spectrophotometer (PERSEE General Instrument Co., Ltd., Beijing, China) at 200–400 nm with a solution of 1 mg/mL in distilled water [20].

The Fourier transform infrared spectroscopy (FT-IR) spectrum was recorded by an infrared spectrometer (Spectrum Two N; PerkinElmer Inc., Waltham, MA, USA) as previously reported [21].

The X-ray diffraction tests were conducted according to Du et al. [22].

The Congo red experiments were conducted according to Zhou et al. [23].

The average particle size and zeta potential of JP (1 mg/mL) were examined at 25 °C using a nanoparticle size potential meter [10].

The apparent viscosity was measured using a rheometer with a polysaccharide concentration of 10 mg/mL. The shear rate was set from 0.01 to 100 s^−1^ [24].

### 2.5. Antioxidant Activity

Referring to the method of Wang et al. [25] and making slight adjustments, the concentration gradient of 1–10 mg/mL of jackfruit polysaccharide (PUAE-JP) was prepared, with vitamin C (VC) as the positive control, to systematically evaluate its in vitro antioxidant activity. The determination indicators included DPPH radical scavenging rate, ABTS radical scavenging rate, hydroxyl radical scavenging rate, and ferric ion reducing ability (FRAP).

### 2.6. Hypoglycemic Activity

#### α-Glucosidase Activity Inhibition Assay

Based on the methods of Jia et al. [26] and Dai et al. [27], and with some adjustments, using acarbose as the positive control, the inhibitory activities of the samples on the two enzymes were determined. α-Glucosidase inhibition experiment: Add 110 μL PBS (0.1 M, pH 6.8), 20 μL sample, and 20 μL α-glucosidase solution (1 U/mL) in a 96-well plate. Incubate at 37 °C for 10 min, then add 20 μL p-NPG (1.25 mM) for 20 min, and finally add 80 μL Na_2_CO_3_ (0.1 M) to terminate the reaction. Measure the absorbance at 405 nm and calculate the inhibition rate according to the formula. α-Amylase inhibition experiment: Add 200 μL sample and 200 μL α-amylase solution (1 U/mL) to a centrifuge tube. Incubate at 37 °C for 10 min, then add 200 μL of 1% soluble starch solution for 20 min, and add 1 mL of DNS reagent. Heat in a boiling water bath for 10 min for color development, then cool down. Measure the absorbance at 540 nm and calculate the inhibition rate according to the formula.

inhibition ratio (%) = [1 − (Absorbance containing the sample and the enzyme − Absorbance of the sample and water)/Absorbance of water and enzymes] × 100%
(1)

### 2.7. In Vitro Fermentation

#### 2.7.1. Preparation of Fecal Inoculum

Fresh feces were collected from 6 healthy volunteers (3 females and 3 males; aged 20–30; without antibiotics or probiotic supplements in the past 3 months; without a history of gastrointestinal diseases; after equal mixing). Fecal suspension was diluted by 0.1 mol/L PBS (pH 7.0) into a concentration of 10% and then filtered through double-layer sterilized gauze to obtain the fecal inoculates [28].

#### 2.7.2. Fermentation

The fermentation medium was prepared according to Zeng et al. [29]. HAE-JP, UAE-JP, and inulin (JF, positive control) were added to the medium at a rate of 1% (*w*/*v*), and the basic nutrient medium containing distilled water was used as the BLANK group. After adjusting the pH of the medium to 7.0 and sterilizing at 121 °C for 15 min, fecal suspension (1 mL) was mixed with medium containing polysaccharides and incubated at 37 °C for 48 h [30].

#### 2.7.3. Analysis of the Intestinal Microbiota Through 16S rRNA Gene Sequencing

Microbial community amplicon sequencing analysis was performed by Guangzhou Genedenovo Biotechnology Co., Ltd. (Guangzhou, China). The specific experimental procedures were as follows: Microbial genomic DNA was extracted using the HiPure Soil DNA Extraction Kit (or HiPure Stool DNA Extraction Kit) according to the manufacturer’s instructions. The V3–V4 hypervariable region of the bacterial 16S rRNA gene was amplified using specific primers under the following thermal cycling conditions: initial denaturation at 95 °C for 5 min; 30 cycles of denaturation at 95 °C for 1 min, annealing at 60 °C for 1 min, and extension at 72 °C for 1 min; and final extension at 72 °C for 7 min. The 50 μL PCR reaction system contained 10 μL of 5 × Q5^®^ Reaction Buffer, 10 μL of 5 × Q5^®^ High GC Enhancer, 1.5 μL of 2.5 mmol/L dNTPs, 1.5 μL of each forward and reverse primer (10 μM), 0.2 μL of Q5^®^ High-Fidelity DNA Polymerase, and 50 ng of template DNA. PCR products were evaluated by 2% agarose gel electrophoresis, purified using AMPure XP Beads (Beckman Coulter, Inc., Brea, CA, USA), and quantified with Qubit 3.0 Fluorometer. Sequencing libraries were constructed using the Illumina DNA Prep Kit (Illumina, San Diego, CA, USA). Library quality was verified using the ABI StepOnePlus Real-Time PCR System (Life Technologies, Foster City, CA, USA). Qualified libraries were pooled and sequenced on the NovaSeq 6000 platform using PE250 mode.

### 2.8. Data Statistics and Analysis

Data analysis and plotting were conducted using Excel 2010 and Origin 2020. One-way analysis of variance and multivariate significance analysis were performed using IBM SPSS Statistics 27 software. The polysaccharide extraction process was optimized and analyzed using Design-Expert 27.0.

## 3. Results

### 3.1. Extraction of Jackfruit Polysaccharide (JP)

The influence of various factors on the yield of JP was shown in Figure S1. From the single-factor experiments, the optimal extraction parameters were solid–liquid ratio 30 g/mL, extraction time 60 min, extraction temperature 70 °C, ultrasonic time 30 min, and ultrasonic power 90 W, respectively. To further optimize the extraction conditions, the response surface optimization (RSM) experiment was conducted. The solid–liquid ratio (A), ultrasonic time (B), and ultrasonic power (C) were selected as independent variables, and the yield of polysaccharides was taken as the response value. RSM results were shown in Tables S2 and S3. The quadratic regression equations was Y = 8.45 + 0.5037A − 0.3120B + 0.2380C − 0.6073AB − 0.1447AC + 0.4668BC − 2.23A2 − 1.04B2 − 0.8499C.

### 3.2. Response Surface Interaction Analysis

**Figure 1:**
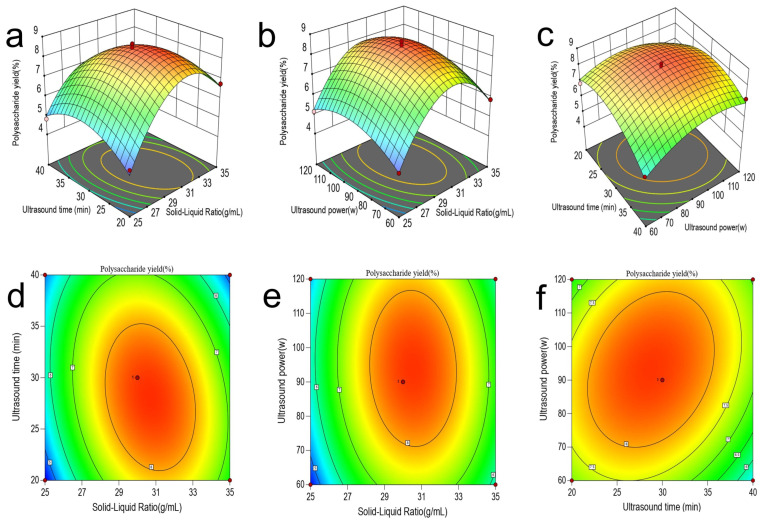
Three-dimensional and contoured maps of the interaction of different variables in the UAE-JP model. (**a**) Three-dimensional surface plot depicting polysaccharide yield as a function of ultrasound time and solid-liquid ratio. (**b**) Three-dimensional surface plot showing the relationship between ultrasound power, solid-liquid ratio, and polysaccharide yield. (**c**) Three-dimensional surface plot representing polysaccharide yield variation with ultrasound power and ultrasound time. (**d**) Contour plot corresponding to panel (**a**), illustrating the interactive effects between ultrasound time and solid-liquid ratio. (**e**) Contour plot corresponding to panel (**b**), demonstrating the interaction between ultrasound power and solid-liquid ratio. (**f**) Contour plot corresponding to panel (**c**), presenting the relationship between ultrasound power and ultrasound time. In all contour plots (**d**–**f**), the color gradient from blue to red indicates increasing polysaccharide yield from low to high, with the central red regions representing optimal parameter combinations for maximum extraction efficiency.

Figure 1 presents the three-dimensional response surface plot and the corresponding contour plots of the pairwise interactions of the solid–liquid ratio (A), the ultrasonic time (B), and the ultrasonic power (C). These are used to analyze the interactions and significant effects among the various factors. By combining Figure 1 with the results of variance analysis (Table S3), it can be seen that the interactions between the solid–liquid ratio and the ultrasonic time (A, B), as well as between the ultrasonic time and the ultrasonic power (B, C), are significant. Specifically, as shown in Figure 1a,c, the slopes of the response surfaces are steep, and the corresponding contour plots (d, f) are clearly elliptical. Meanwhile, the *p*-values of the corresponding items in Table S3 are all less than 0.05. In contrast, the response surface in Figure 1b is relatively flat, and the corresponding contour plot (e) deviates from the elliptical shape, indicating that the interaction between the solid–liquid ratio and the ultrasonic power (A, C) is not significant. This result is consistent with the statistical conclusion in Table S3, where the corresponding *p*-values are greater than 0.05.

### 3.3. Optimization and Validation Results of the Model

The model determined the optimal technological parameters for UAE-JP as the solid–liquid ratio of 30.6697 mL/g, ultrasonic time of 28.2869 min, and ultrasonic power of 92.4301 W (Figure 1). The model predicted that the yield of polysaccharides was 8.516%. For the convenience of the extraction operation, the process parameters were adjusted to a liquid-to-material ratio of 30 mL/g, an ultrasonic time of 30 min, and an ultrasonic power of 90 W. The adjusted process parameters were verified by experiments. After three groups of parallel experiments, the yield of JP was 8.70 ± 0.11%, which was close to the predicted value (8.516%). This high degree of consistency indicates that the model is applicable to practical extraction applications.

To evaluate the reliability of the response surface model constructed in this study and the effectiveness of the ultrasound-assisted extraction (UAE) process, a systematic comparison was conducted with similar process models reported by Wu et al. [31]. In terms of model performance, the model developed in this study (Table S3) exhibited higher goodness-of-fit (R^2^ = 0.9870, RAdj^2^ = 0.9704) and superior predictive stability (RAdj^2^ − RPred^2^ = 0.1209), outperforming the comparative model (R^2^ = 0.9741, adjusted R^2^ = 0.9483). Regarding process performance, compared to the traditional hot water extraction method (90 °C, 2.5 h, yield 6.18%) [9], the optimized UAE process in this study (30 min, 90 W) reduced the extraction time by 80% while increasing the polysaccharide yield to 8.70% (an increase of approximately 41%). In contrast to microwave-assisted extraction, UAE eliminates the need for highly acidic media (pH 1.0–2.0) and precise temperature control (65.99 °C) [32], thereby reducing the risk of local overheating that could damage polysaccharide structures. Compared to enzyme-assisted extraction (cellulase addition 5.64%, 55.65 °C, 3.4 h, pH 5.22) [33], the UAE process does not rely on exogenous enzymes, simplifying the operational workflow, reducing costs, and avoiding the additional purification burden that may arise from residual enzymes. In summary, the model developed in this study demonstrates high reliability, and the corresponding UAE process exhibits clear advantages in terms of extraction efficiency, operational simplicity, mild conditions, and cost-effectiveness, providing a viable and practical technical solution for the efficient and green extraction of jackfruit polysaccharides.

### 3.4. Isolation and Purification of JP

**Figure 2:**
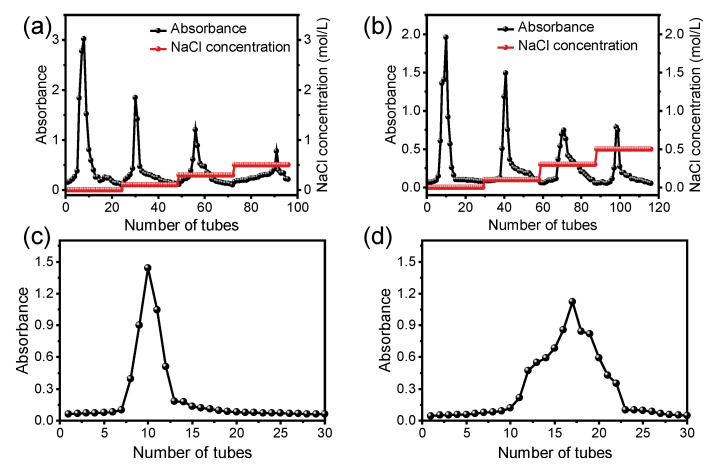
Elution curves of HAE-JP (**a**) and UAE-JP (**b**) on a DEAE cellulose-52 column; elution curves of HAE-JP (**c**) and UAE-JP (**d**) on glucan G-100. In panels (**c**,**d**), the black dots represent experimental absorbance measurements at 490 nm collected from consecutive fractions (tubes), and the solid black line connects these points to illustrate the elution profile of the polysaccharide fraction purified with distilled water.

When purifying the polysaccharides of jackfruit, DEAE-52 was first used for stepwise elution with salt solutions of different concentrations. Finally, four polysaccharide fractions were obtained, and the elution curves are shown in Figure 2. Since the yield of the water-eluted fraction was higher than that of the NaCl-eluted fraction, we collected the water-eluted fraction for secondary purification. The freeze-dried water-eluted fraction was purified by a Sephadex G-100 column, and the higher absorbance fraction was collected as purified JPs for further characterization.

### 3.5. Physicochemical Properties of JPs

#### 3.5.1. Chemical Composition

**Figure 3:**
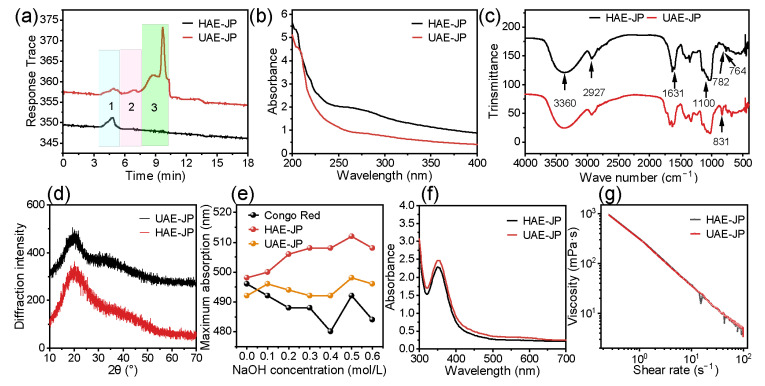
Molecular weight distribution (**a**),1–3, polysaccharide fractions with different molecular weights. UV–Vis absorption spectrum (**b**). FT-IR spectrum (**c**). X-ray diffraction pattern (**d**). Maximum absorption wavelength of polysaccharide-carmine complex at different NaOH concentrations (**e**). UV–visible absorption spectrum of polysaccharide in jackfruit flesh after reaction with iodine reagent (**f**). Shear rate dependence flow curves (**g**).

As shown in Table 1, the yield of UAE-JPs (8.70%) was significantly higher than that of HAE-JPs (5.95%), consistent with the research results of Wei et al. [34]. This was ascribed to the cavitation, mechanical, and thermal effects of ultrasonication, which could effectively destroy the cell wall and promote the release of polysaccharides [35]. The uronic acid content of UAE-JPs (15.47%) was also higher than that of HAE-JPs (12.78%), suggesting that the acidic polysaccharides were more abundant. This was consistent with the results of the monosaccharide composition analysis (Table 2), and this difference was due to the fact that the ultrasonic cavitation effect can effectively destroy the cell wall, thereby facilitating the complete release and decomposition of acidic polysaccharides [36]. HAE-JPs and UAE-JPs are both negatively charged anionic polysaccharides with good stability. Among them, the zeta potential and particle size of UAE-JPs were lower than those of HAE-JPs. Previous studies had confirmed that polysaccharides with lower zeta potential and smaller particle size usually depicted stronger antioxidant activity [23,26]. This phenomenon was attributed to the cavitation effect produced by ultrasonic high-frequency vibration and the strong mechanical shear force. On the one hand, it promoted the dissociation of the polysaccharide aggregates, resulting in a reduction in particle size; on the other hand, by exposing more charged groups (such as the carboxyl group of glucuronic acid), the zeta potential is reduced [37]. The molecular weight of HAE-JPs varied greatly (293.65 × 10^4^–0.37 × 10^4^ Da), while that of the UAE-JPs was concentrated around 86.60 × 10^4^ Da and 2.64 × 10^4^ Da, indicating that ultrasound could promote the degradation of macromolecules and make the molecular weight distribution more concentrated (Figure 3a). Similar phenomena were also observed in previous studies [38]. The enhanced bioactivity of low-molecular-weight polysaccharides can be attributed to their increased exposure of free hydroxyl groups, elevated reducing sugar content, and improved accessibility of active sites [39].

Monosaccharide composition analysis (Table 2, Figure S2) revealed that jackfruit polysaccharide (JP) is a heteropolysaccharide composed of 13 monosaccharides, confirming its structurally complex nature. D-Glucose was identified as the primary component, with molar percentages of 59.71% and 62.20% in HAE-JP and UAE-JP, respectively. The most significant structural change induced by ultrasound-assisted extraction (UAE) was the specific enrichment of the acidic monosaccharide D-galacturonic acid (GalA). The molar percentage of GalA in UAE-JP (10.28%) was 6.3 times that in HAE-JP (1.62%), and its absolute content increased by 11.52-fold (7.54 → 86.90 mg/g). This finding is consistent with the report by Li et al. [40], who observed that ultrasonic treatment significantly increased the galacturonic acid content (8.7% → 12.3%) and antioxidant activity of red raspberry pectic polysaccharides. The enrichment of this acidic sugar, along with the novel detection of D-xylose (1.04%) exclusively in UAE-JP (not detected in HAE-JP), indicates that ultrasonic cavitation effectively disrupts the cell wall structure, thereby facilitating the release of acidic polysaccharide fragments closely associated with pectin and hemicellulose. Concurrently, the molar percentages of some neutral monosaccharides (e.g., D-galactose, L-arabinose, and L-rhamnose) showed a relative decrease alongside the substantial increase in total acidic sugar content, supporting the view that ultrasonic treatment primarily remodels the molar ratios rather than the types of monosaccharides [41]. Structural parameter analysis further elucidated conformational differences. The rhamnose/galacturonic acid (Rha/GalA) molar ratio of UAE-JP (0.53) was significantly lower than that of HAE-JP (4.39), indicating a substantially higher proportion of linear homogalacturonan (HG) regions and a more linearized overall structure in UAE-JP. In contrast, the (Arabinose + Galactose)/Rhamnose [(Ara + Gal)/Rha] ratio of HAE-JP (3.64) was higher than that of UAE-JP (3.15), also supporting the inference of a higher degree of branching in HAE-JP [42]. In summary, UAE is a technique capable of directionally modifying the fine structure of polysaccharides by specifically enriching acidic sugars, releasing xylose units, and reducing the overall degree of branching, thereby simplifying the linear structure. These structural alterations provide a crucial material basis for explaining the enhanced antioxidant activity and potential differential prebiotic functions of UAE-JP.

#### 3.5.2. Results and Discussion of Structural Characterization

There were no significant absorption peaks at wavelengths of 260 nm and 280 nm (Figure 2b), suggesting that the protein and nucleic acid were extremely low in JP, consistent with the chemical composition (Table 1).

Figure 3c was the FT-IR spectrum of JP. The broad peak at 3360 cm^−1^ was an O-H stretching vibration, indicating that JP was rich in hydroxyl groups and had hydrogen bonding interactions [43]. The absorption capacity of UAE-JP was stronger than that of HAE-JP. This difference may be related to the fact that ultrasonic treatment promotes the formation or exposure of more hydroxyl groups (O-H groups) [44]. The stretching vibration of C-H at 2927 cm^−1^ confirmed the saturated hydrocarbon chain skeleton [45]. The C=O vibration at 1631 cm^−1^ was the peak of uronic acid [46]. The peak absorption intensity at 1631 cm^−1^ in UAE-JP was greater than that in HAE-JP, which was consistent with our analysis of the monosaccharide composition (Table 2). The peak between 1000 and 1100 cm^−1^ was associated with the C-O-C glycosidic bond and the C-O-H vibration, confirming the presence of a pyranose ring in the main chain [47]. The peak at 831 cm^−1^ was attributed to the α-configuration, and the peaks at 764 cm^−1^ (HAE) and 782 cm^−1^ (UAE) confirmed the symmetrical conformation of the pyran sugar ring [48]. The results showed that HAE-JP and UAE-JP contained uronic acid, pyranose, and α-glycosidic bonds. The functional groups of JP extracted by the two methods were similar, indicating that the ultrasonic-assisted extraction did not significantly affect the chemical structure of the polysaccharides.

As shown in Figure 3d, there were no sharp diffraction peaks in the XRD patterns of both HAE-JP and UAE-JP, indicating the amorphous structures of polysaccharides, which were similar to the result of jackfruit polysaccharide reported by Yu et al. [10]. The broadening of the UAE-JP diffraction peaks indicates that the crystal grains of the polysaccharide may have decreased. This result was consistent with the research conclusion of Mansour et al. [49].

Figure 3e was the result of the Congo red experiment. Both UAE-JP and HAE-JP showed a significant red shift when adding NaOH, indicating that ultrasonic treatment did not destroy the triple helix structure [50]. When NaOH concentration was 0.5 M, both HAE-JP and UAE-JP depicted the largest red shift of 14 nm and 6 nm, respectively. The difference in red shift might be due to the degradation of polysaccharides and different hydrogen bond components [51]. The iodine-potassium iodide experiment (Figure 3f) showed that both HAE-JP and UAE-JP exhibited distinct absorption peaks at 350 nm. This characteristic peak indicates that JP belongs to a polysaccharide with a highly branched structure, and the ultrasonic treatment did not alter its complex branched structure. This conclusion was consistent with the research findings of Zhang et al. [52].

JP depicted non-Newtonian shear-thinning behavior (Figure 3g), which was mainly attributed to the untanglement of molecular chains [53]. At the same shear rate, the apparent viscosity of UAE-JP was slightly lower than that of HAE-JP. The strong mechanical effect of ultrasound not only broke the molecular chains but also formed a looser linear structure. This structure was more likely to be oriented and arranged under shear force, reducing the intermolecular tangled effect and further reducing the viscosity.

In this study, the polysaccharides extracted from durian by ultrasound/water heating (HAE-JP → UAE-JP) exhibited systematic structural differences from the polysaccharides obtained through lactic acid bacteria fermentation of durian (JP → JP-F) [10], revealing the differential regulatory mechanisms of “physical extraction modification” and “biological fermentation modification” on the structure of polysaccharides. In terms of physicochemical properties, ultrasound treatment significantly reduced the molecular weight of the polysaccharides (from 293.65 × 10^4^ Da to 86.60 × 10^4^ Da), specifically enriching glucuronic acid (from 7.54 to 86.9 mg/g) and showing higher solubility (80.67%); while the fermentation treatment could also reduce the molecular weight (from 320.21 KDa to 243.62 KDa) and increase solubility (64.87%), the content of glucuronic acid did not show significant enrichment. At the single sugar composition level, UAE-JP exhibited the characteristic of “acidic monosaccharide (such as glucuronic acid) enrichment”, while JP-F showed a “neutral monosaccharide reconfiguration” pattern with an increase in the ratio of arabinose and galactose and a decrease in the ratio of glucose. Although there are significant differences in the primary structures, such as molecular weight and single sugar composition, between the two, infrared spectroscopy analysis indicated that they both retained the typical functional group configurations (such as characteristic absorption peaks of O-H, C-H, C=O, etc.) and amorphous structure characteristics of polysaccharides.

### 3.6. In Vitro Antioxidant Activity of JP

**Figure 4:**
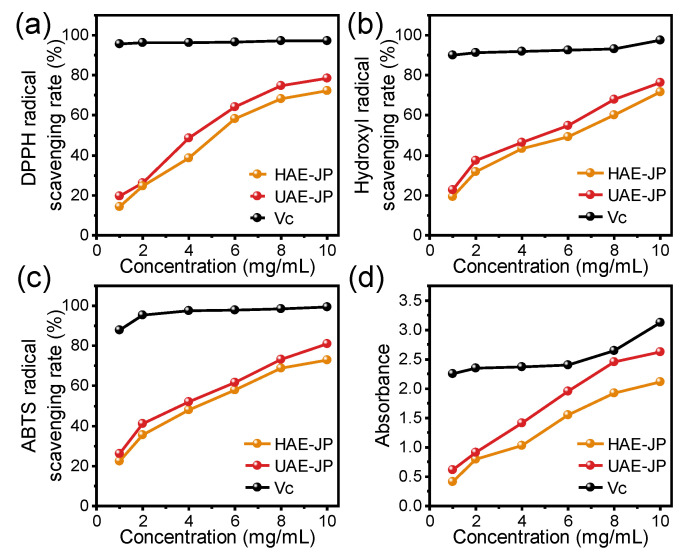
Analysis of antioxidant capacities for JP. DPPH (**a**), hydroxyl (**b**), and ABTS (**c**) radical scavenging activities. Ferric reducing antioxidant ability (**d**).

The research by Zhu et al. [54] has confirmed that the polysaccharides in the flesh of jackfruit showed significant antioxidant activity. In this study, four indicators, namely DPPH, ABTS, hydroxyl radical scavenging rate, and iron ion reduction ability (FRAP), were adopted to systematically evaluate the antioxidant activity of the samples (Figure 4). Within the concentration range of 0–10 mg/mL, the antioxidant capacity of the HAE-JP and UAE-JP was increased in a dose-dependent manner, which was consistent with other research on the antioxidant capacity of vegetable polysaccharides [55]. The half-inhibitory concentration (IC_50_) of DPPH free radicals for HAE-JP and UAE-JP was 5.86 and 4.89 mg/mL, respectively. For ABTS free radicals, the IC_50_ was 5.01 and 4.15 mg/mL, respectively. For hydroxyl free radicals, the IC_50_ was 5.93 and 4.99 mg/mL, respectively. These data indicated that the free radical scavenging ability of UAE-JP was significantly stronger than that of HAE-JP. This discovery was consistent with the research results of Xu et al. Additionally, UAE-JP depicted a stronger reductant ability, but both are much lower than ascorbic acid (Figure 4d). You et al. [56] also found that the platelet polysaccharides extracted by ultrasound had enhanced iron-removing ability. Ultrasound treatment causes the breaking of glycosidic bonds in polysaccharides, significantly reducing the molecular weight of UAE-JP (Table 1). This decrease in molecular weight not only increases the number of reducible terminal hydroxyl groups that can directly eliminate free radicals [57], providing more reaction targets for free radicals, but also significantly enhances the water solubility and specific surface area of UAE-JP, optimizing its contact efficiency with free radicals, thereby strengthening the kinetics of the antioxidant reaction. Moreover, UAE-JP contains higher amounts of acidic monosaccharides (such as galacturonic acid) (Table 2). The carboxyl groups (-COOH) in its molecular structure can quickly capture and quench free radicals through efficient proton transfer or electron transfer mechanisms, further enhancing the antioxidant efficacy [58]. Additionally, glucuronic acid can chelate Fe^2+^, inhibit the generation of free radicals, and alleviate oxidative stress [56]. In conclusion, the results of this study confirm that the antioxidant activity of polysaccharides is a function of their multi-dimensional structural characteristics. Besides molecular weight, the composition of monosaccharides is also a key parameter determining their activity, which is consistent with the view of Janardhanan et al. [59] regarding the relationship between polysaccharide structure and activity.

To more clearly evaluate the antioxidant activity level of jackfruit polysaccharides (JP), this study compared their activity data with that of polysaccharides from other natural sources reported in the literature. At the same testing concentration (5 mg/mL), red raspberry pectin extracted by UAE showed DPPH and hydroxyl radical scavenging rates of 57.23% and 59.52% [60], respectively, slightly higher than those of UAE-JP in this study (50.75% and 50.04%). Compared to the polysaccharide fraction extracted from Pleurotus eous by Janardhanan et al. [59] (DPPH IC_50_ = 16.21 mg/mL, ABTS IC_50_ = 16.06 mg/mL), JP exhibited significantly stronger free radical scavenging capacity. Additionally, compared to the most active purified fraction, BMP1-1 from black mulberry fruit, reported by Wang et al. [61], at a concentration of 4 mg/mL, its ABTS and hydroxyl radical scavenging rates were 50.62% and 4.16%, respectively, while the corresponding scavenging rates of UAE-JP in this study at the same concentration were 52.05% and 46.47%, indicating superior overall antioxidant activity. Overall, mangosteen polysaccharides demonstrated moderate to above-average antioxidant capacity and possess good development potential.

### 3.7. In Vitro Hypoglycemic Activity of JP

**Figure 5:**
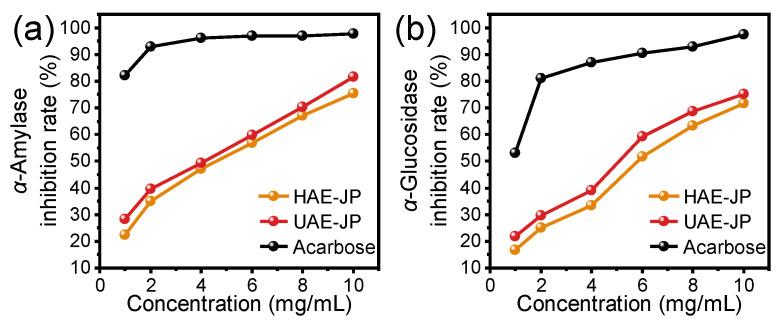
Antidiabetic activity JP. Inhibition rate of α-amylase (**a**) and α-glucosidase (**b**) activity. Acarbose was used as a positive control.

The inhibition rates of JP on α-amylase and α-glucosidase were increased in a dose-dependent manner (Figure 5). The IC_50_ of HAE-JP and UAE-JP on α-glucosidase inhibitory activity were 6.19 and 5.33 mg/mL, respectively, while their IC_50_ values against α-amylase were 5.04 and 4.31 mg/mL, respectively. Studies have shown that the α-glucosidase inhibitory activity of polysaccharides was closely related to their structural characteristics. Besides the main branched structure, polysaccharides rich in glucose components with a triple helix structure could significantly increase the α-glucosidase inhibitory rate [62]. The higher inhibitory activity against α-glucosidase of UAE-JP might be related to its higher glucose molar percentage (62.2%) (Table 2) and potential triple helix structure (Figure 3f). This characteristic was consistent with the structural and activity relationship pattern of the dried longan polysaccharide extracted by ultrasound-assisted extraction [63], further confirming the regulatory effect of the extraction method on the structure and functional activity of polysaccharides. Although the inhibition rates of the two enzymes by JP were slightly lower than those of the positive control, acarbose, JP still depicted potential as an anti-diabetic drug.

### 3.8. The Influence of JP on Intestinal Flora

#### 3.8.1. Analysis of the Results of in Vitro Fermentation

Figure 5a depicts the fermentation process of different samples of intestinal flora. As the fermentation time increases, the density of each group of fermentation liquid rises. The FJ group, due to no substrate limitation, showed a continuously increasing OD_600_, and the microbial proliferation was active. When JP was used as the sole carbon source for fermentation, the total biomass of the microbial community in the fermentation liquid showed a gradually increasing trend. During the fermentation, the OD_600_ values of both UAE-JP and HAE-JP were higher than the positive control of inulin, indicating that JP can significantly increase the bacterial density. These results suggested that the polysaccharides obtained by the two extraction methods can be better utilized by the intestinal microbiota.

#### 3.8.2. Species Composition

**Figure 6:**
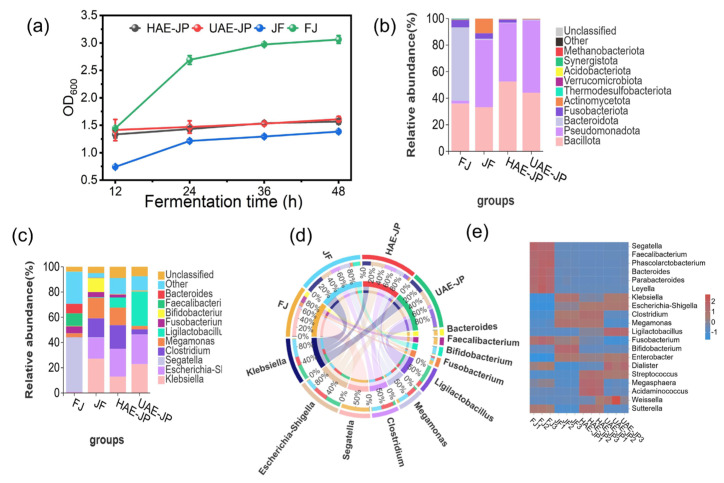
OD_600_ of in vitro fermentation (**a**). Analysis of species composition. Phylum level (**b**), genus level (**c**), genus-level Circos (**d**), and genus-level heatmap (**e**). FJ: the blank control (no addition of carbon source supplement), JF: inulin (positive control).

At the phylum level (Figure 6b), the dominant bacterial phyla in FJ were *Bacteroidota* (55.32% ± 2.41%), *Bacillota* (35.94% ± 2.31%, formerly known as *Firmicutes*), and *Fusobacteria* (5.45% ± 0.46%). This was consistent with the previous research results [64]. The abundance of *Bacillota* in UAE-JP and HAE-JP was 52.49% ± 0.38% and 44.05% ± 1.71%, higher than that in FJ, respectively. *Bacillota* could produce beneficial short-chain fatty acids (SCFAs) for host health by fermenting polysaccharides. The relative abundance of the Fusobacterium genus associated with cancer was significantly reduced in HAE-JP, UAE-JP, and JF, decreasing by 3.08% ± 0.61%, 5.23% ± 0.64%, and 1.30% ± 0.77%, respectively [65]. Additionally, the abundance of Bacteroidota in HAE-JP, UAE-JP, and JF groups was lower than that in the control group, consistent with the results of Moon et al. [66]. Based on the structural characteristics of polysaccharides, UAE-JP has more acidic sugars (such as galacturonic acid) exposed due to ultrasonic extraction (Table 1). HAE-JP retains a considerable amount of long-chain neutral sugars but still has certain acidic groups. These structural characteristics may affect the pH value of the local intestinal microenvironment. Moreover, some studies have indicated that a low pH environment was conducive to the growth of the Firmicutes phylum but inhibited the growth of the Bacteroidetes phylum [67].

At the genus level (Figure 6c), the FJ group mainly consisted of *Segatella* (43.11% ± 2.44%), *Faecalibacterium* (9.98% ± 0.25%), and *Bacteroide* (7.55% ± 0.45%). Compared with the FJ group, the HAE-JP, UAE-JP, and JF groups significantly increased the abundance of *Clostridium* and *Megamonas*. Among them, the increase in HAE-JP and JF groups was more prominent. The higher increase in HAE-JP may be related to its branched structure diversity; complex structures provide diverse carbon sources. Combined with the research of Wu et al. [68] (high branching degree enhances the regulatory effect of intestinal microorganisms), it was speculated that these genus groups could achieve a proliferation advantage by specifically metabolizing their branched structures. At the same time, the abundance of *Ligilactobacillus* in the HAE-JP and UAE-JP groups significantly increased (7.94% ± 0.16% and 27.04% ± 0.889%, respectively), which was much higher than that of the FJ group (0.1%). The abundance of *bifidobacteria* in the JF group and the UAE-JP group also increased. These results confirmed the prebiotic activity of polysaccharides by selectively enriching beneficial bacteria such as *Ligilactobacillus* and *bifidobacteria* to regulate the intestinal microecology, which was consistent with the conclusion of the study on intracellular polysaccharides of Chlorella [69]. The complex branching structure provides diverse enzyme action sites for butyrate-producing bacteria (such as bacteria from the Clostridia order). These bacteria encode a wide range of glycoside hydrolases that can precisely recognize and degrade the glycosidic bonds at the branching points. This slow and complex degradation process not only creates a stable ecological niche for the relatively slow-growing butyrate-producing bacteria, but also generates more fermentation precursors after the decomposition of its complex carbon framework structure, ultimately leading to higher butyrate production [70]. In contrast, the utilization of low-molecular-weight UAE-JP conforms to the “enzyme system adaptability” principle [71]. Its small molecule and high solubility characteristics enable it to be rapidly absorbed without the need for a complex enzyme system. Bacterial groups such as lactic acid bacteria and *bifidobacteria*, with their efficient oligosaccharide transport systems [72], can preferentially take in and rapidly ferment these low-molecular-weight fragments, mainly producing lactic acid and acetic acid through homofermentative or heterofermentative fermentation. This process rapidly lowers the local pH in the intestine, and the formed acidic environment inhibits some harmful bacteria while further strengthening the competitive advantage of these probiotics, promoting their rapid proliferation.

It was worth noting that adding JP reduced the abundances of *Fusobacterium*, Enterobacter, and *Bacteroides* species. *Fusobacterium* was associated with bone cancer and intestinal inflammation, which suggested that the JP may have potential anti-tumor or anti-inflammatory effects. The abundances of *Escherichia-Shigella* and *Klebsiella* species increased, which may be related to the fact that these bacterial groups are more capable of utilizing low-molecular-weight carbon sources [73].

In summary, UAE-JP had better promotion than HAE-JP in the growth of rapid-fermenting probiotic species such as *Lactobacillus* and *Bifidobacterium*, and was suitable for short-term regulation of the acidic environment in the intestinal tract. HAE-JP was more likely to promote the proliferation of butyrate-producing bacteria (such as *Clostridium*), and might have an advantage in maintaining intestinal homeostasis (anti-inflammatory, barrier repair, etc.) in the long term. JF showed a different regulatory pathway from HAE-JP and UAE-JP, which might be related to the fact that its polysaccharides contained more oligosaccharide components. UAE-JP, HAE-JP, and JF could improve the intestinal microecology through different mechanisms.

Figure 6d depicts the significant differences in the proportion of different bacterial genera. The FJ sample was dominated by *Segatella*. In the HAE-JP sample, the proportions of *Clostridium* and *Megamonas* are prominent, and the proportion of *Ligilactobacillus* (lactic acid bacteria genus) increased. The *Bifidobacterium* genus had an increased proportion in the JF sample. In the UAE-JP sample, *Ligilactobacillus* became the dominant genus.

The heatmap of abundance at the genus level in Figure 6e further confirmed that the microbial composition varies. *Segatella* showed a relatively high abundance in the FJ sample, while the JF group was mainly characterized by high abundances of *Bifidobacterium*. In the group treated with JP, the abundances of beneficial bacteria (*Megamonas*, *Dialister*, *Weissella*, *Ligilactobacillus*) increased, while the abundances of harmful bacteria (*Fusobacterium*) decreased. Moreover, the effect of JP on the intestinal microbiota varies depending on the extraction method. In the HAE-JP group, the abundances of *Megasphaera*, *Acidaminococcus*, *Megamonas*, and *Clostridium* increased. In the UAE-JP group, *Dialister*, *Weissella*, and *Ligilactobacillus* were significantly enriched. Overall, these findings indicate that supplementing JP could change the composition of the intestinal microbiota in a structure-dependent manner and is expected to play a positive role in maintaining intestinal homeostasis. This result was consistent with the conclusion of the study by Wan et al. [74].

#### 3.8.3. Alpha Diversity Analysis

**Figure 7:**
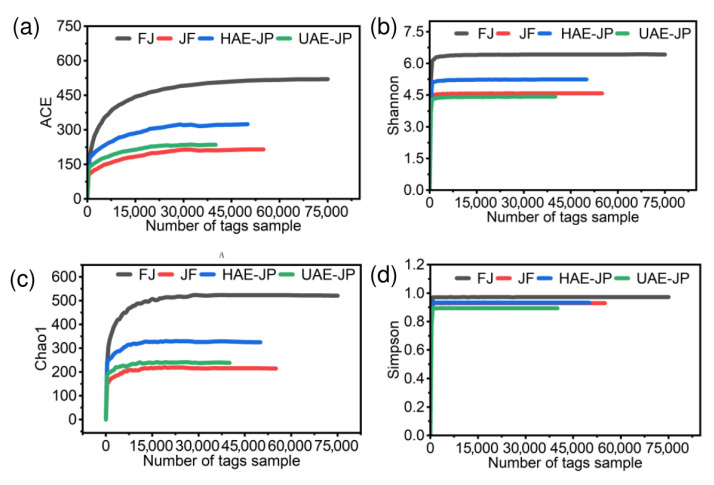
The sparse curve of intestinal flora. ACE index (**a**), Shannon index (**b**), Chao1 index (**c**), and Simpson index (**d**).

Figure 7 shows the sparse curves of each group of samples, with the index types being Shannon, Chao1, Simpson, and ACE. With the increase in sequencing data volume, each index curve reached a plateau stage, indicating that sufficient sequencing depth had been obtained to cover the true diversity of microbial species in the samples, and the data volume met the requirements for subsequent analysis [75]. The Shannon and Simpson indices reflected the diversity of the intestinal flora (higher values indicate greater diversity), while the ACE and Chao indices are positively correlated with richness (larger values indicate higher richness) [69].

Table 3 showed that there were differences in the effects of different groups on the richness and diversity of the intestinal microbiota. The richness of the community was in the order of FJ > HAE-JP > UAE-JP > JF, and the diversity was in the order of FJ > HAE-JP > JF > UAE-JP. This difference may be related to the extraction method. Thermal extraction (HAE) retained more long-chain neutral sugars and intact active fragments, which was conducive to maintaining high microbial richness. Ultrasonic extraction (UAE) exposed acidic sugars and changed the intestinal metabolic environment, resulting in a decrease in diversity, which was consistent with the results of monosaccharide composition in Table 2. However, compared with the FJ group, the intestinal microbiota in the JF, HAE-JP, and UAE-JP groups showed a decrease in both quantity and diversity (Table 3). This decline might be due to the addition of JF, HAE-JP, and UAE-JP, which led to an increase in the number of specific microorganisms, and the competitive growth of dominant bacterial groups might inhibit the colonization of other microbial groups, resulting in a tendency towards ‘exclusive enrichment’ of the microbiota structure, thereby manifesting a decrease in overall quantity and diversity [43]. Specifically, the inclusion of UAE-JP resulted in a significant proliferation of beneficial bacterial strains such as *Bifidobacterium*, demonstrating a positive prebiotic effect. However, it was also accompanied by an increase in potential pathogenic bacterial strains such as *Escherichia-Shigella*, which brings certain warning significance. This makes the overall impact of this on intestinal health more complex and may have strain-specific differences. Therefore, to clearly understand the balance of benefits and drawbacks of this effect, future research should use metagenomic sequencing to identify specific species and analyze their functional genes to obtain more accurate assessment results. This was consistent with previous research for polysaccharides from *Lactiplantibacillus* plantarum T1 [76] and shiitake mushrooms [77].

## 4. Conclusions

This study systematically revealed the mechanism by which ultrasound-assisted extraction (UAE) achieves efficient extraction and structural modification of jackfruit polysaccharides (JP) through cavitation effects. While the UAE, by destroying the cell walls, achieved an extraction rate of 8.70% (2.75% higher than that of the traditional hot water extraction method), it modifies the molecular characteristics of JP: the large molecular weight component decreases from 293.65 × 10^4^ Da to 86.60 × 10^4^ Da, the particle size reduces from 1621.67 nm to 644.30 nm, and it increases the degree of esterification and uronic acid content, enhancing the accessibility of acidic monosaccharide sites. Structure–activity relationship analysis indicates that the structural modifications induced by UAE are closely related to the enhanced biological activity of JP. The increased exposure of acidic monosaccharide sites and improved molecular weight compatibility jointly strengthen its role as a functional active center, making UAE-JP significantly superior to HAE-JP in DPPH and ABTS radical scavenging capacity, as well as α-amylase and α-glucosidase inhibitory activity. The two types of JP show significant differences in regulating the intestinal microbiota: UAE-JP, with its appropriate molecular weight, high degree of esterification, and strong accessibility of acidic sites, is more easily utilized by microorganisms, mainly promoting the proliferation of fast-growing probiotics such as *Lactobacillus*, rapidly producing acid to inhibit pathogenic bacteria and achieving short-term microecological regulation; while HAE-JP, with its stable structure and slow fermentation, mainly enriches butyrate-producing bacteria such as *Clostridium* (relative abundance increased by 18.56%), which is beneficial for maintaining long-term intestinal homeostasis and barrier function. These results indicate that UAE-JP has the potential to be used as a high-efficiency prebiotic and functional food ingredient. However, it should be noted that this study still has certain limitations: the in vitro intestinal model cannot fully simulate the human internal environment, and the actual physiological effects need to be verified through animal experiments; the lack of nuclear magnetic resonance and other fine structural analysis methods limits the in-depth analysis of sugar chain connection modes. Future research will combine animal experiments, nuclear magnetic resonance, and multi-omics analysis to further clarify its structure-activity mechanism and promote the development of functional foods.

## Figures and Tables

**Table 1:** Chemical composition, ζ-potential, and particle size of JPs.

		HAE-JP	UAE-JP
Extraction yield (%)		5.95 ± 0.1%	8.70 ± 0.11%
Total sugar (%)		78.68 ± 1.31%	70.31 ± 2.13%
Protein (%)		0.67 ± 0.16%	0.17 ± 0.01%
Uronic acid (%)		12.78 ± 0.64%	15.47 ± 0.26%
Solubility (%)		71.03 ± 2.71%	80.67 ± 2.21%
DE (%)		53.37 ± 2.96%	62.40 ± 2.08%
*ζ*-potential (mv)		−23.27 ± 0.42	−22.30 ± 1.06
Particle size (nm)		1621.67 ± 219.24	644.30 ± 22.70
Molecular weight × 10^4^ (Da)	Peak 1	293.65	86.60
	Peak 2	13.29	15.08
	Peak 3	0.37	2.64

The values shown in the figure are the mean ± standard deviation.

**Table 2:** Monosaccharide composition of polysaccharides.

	Molar Percentage %		Monosaccharide Content mg/g		Fold-Change (UAE-JP/HAE-JP)
Sample Name	HAE-JP	UAE-JP	HAE-JP	UAE-JP	
L-glucuronic acid	0.33	0.13	1.56	1.06	0.68 (↓)
D-mannuronic acid	0.36	0.07	1.67	0.55	0.33 (↓)
D-mannose	0.96	0.89	4.16	7.00	1.68 (↑)
D-glucosamine	0.95	0.28	4.92	2.61	0.53 (↓)
D-ribose	0.13	0.12	0.45	0.76	1.69 (↑)
L-rhamnose	7.11	5.45	28.07	38.93	1.39 (↑)
D-glucuronic acid	1.86	1.83	8.66	15.43	1.78 (↑)
D-galacturonic acid	1.62	10.28	7.54	86.90	11.52 (↑)
D-glucosamine	0.27	0.24	1.40	2.21	1.58 (↑)
D-glucose	59.71	62.20	258.62	487.92	1.89 (↑)
D-galactose	17.02	10.21	73.72	80.09	1.09 (↑)
D-xylose	-	1.04	0.00	6.78	-
L-arabinose	8.86	6.92	31.98	45.25	1.41 (↑)
L-fucose	0.82	0.36	3.23	2.60	0.80 (↓)

The “Fold-Change (UAE-JP/HAE-JP)” column represents the ratio of monosaccharide content in UAE-JPs to that in HAE-JPs; “↑” indicates an increase in content in UAE-JPs compared to HAE-JPs, while “↓” indicates a decrease. “-” indicates that the monosaccharide was not detected in the corresponding sample; D-xylose was newly detected in UAE-JPs.

**Table 3:** Alpha diversity index comparison based on the total number of OTUs.

Groups	ACE	Chao1	Shannon	Simpson
FJ	520.67 ± 36.23	520.67 ± 36.23	6.43 ± 0.08	0.97 ± 0.00
JF	214.67 ± 4.16	214.67 ± 4.16	4.58 ± 0.02	0.93 ± 0.00
HAE-JP	325.00 ± 25.24	325.00 ± 25.24	5.24 ± 0.04	0.93 ± 0.00
UAE-JP	237.00 ± 58.51	237.00 ± 58.51	4.42 ± 0.08	0.89 ± 0.00

## Data Availability

The data presented in this study are available from the corresponding authors upon reasonable request due to legal restrictions.

## Supplementary Materials

The following supporting information can be downloaded at: https://www.mdpi.com/article/10.3390/foods15010132/s1. Table S1. Box-Behnken response surface experimental factor levels; Table S2. Response surface test results; Table S3. Variance analysis of the model; Figure S1. Influence of different factors and levels on the yield of polysaccharides. (a) Solid-liquid ratio, (b) extraction time, (c) extraction temperature, (d) ultrasonic time, (e) ultrasonic time. Different letters (a, b, c, and d) indicate significant differences (n = 3, *p* < 0.05); Figure S2. Monosaccharide composition chromatograms of jackfruit polysaccharides (JP); (a) Chromatogram of a standard mixture of 14 monosaccharides; (b) Acid hydrolysate of polysaccharides obtained by hot water extraction (HAE-JP); (c) Acid hydrolysate of polysaccharides obtained by ultrasound-assisted extraction (UAE-JP); the numbered peaks (1–14) in the standard chromatogram (a) correspond to:1 L-glucuronic acid; 2 D-mannuronic acid; 3 D-mannose Man; 4 D-glucosamine GlcN; 5 D-ribose Rib; 6 L-rhamnose Rham; 7 D-glucuronic acid GlcUA; 8 D-galacturonic acid GalUA; 9 D-glucosamine GalN; 10 D-glucose Glc; 11 D-galactose Gal; 12 D-xylose Xyl; 13 L-arabinose Ara; 14 L-fucose Fuc.

## Abbreviations

The following abbreviations are used in this manuscript:

JP	Jackfruit polysaccharide
HAE-JPs	Heat-assisted extraction of jackfruit polysaccharides
UAE-JPs	Ultrasound-assisted extraction of jackfruit polysaccharides
DE	Degree of esterification
