# Supplementary material for: Effect of Ultrasonic-Assisted Extraction on the Structural and Physiological Activity of Jackfruit Polysaccharides
Source: Foods. 2026 Jan 2;15(1):132. doi: 10.3390/foods15010132 (PMC12785949; doi:10.3390/foods15010132)
Supplement: Supplementary file 1 [file foods-15-00132-s001.zip › foods-4032607-supplementary.pdf]

foods

Supporting Information

Effect of Ultrasonic-Assisted Extraction on the Structural and Physiological Activity of Jackfruit Polysaccharides

Jinmei Hu [1,3], Zongcheng Luo [1], Fengzhen You [1], Donghui Luo [1,2,3], Fengchuan Ma [1], Zhong-Sheng Tang [1,2,3]* and Siming Zhu [1,4]**

[1] College of Food Science and Engineering, Guangdong Ocean University, Yangjiang, Guangdong
[2] Yangjiang Research Institute of Guangdong Ocean University, Yangjiang, Guangdong
[3] Chaozhou Branch of Chemistry and Chemical Engineering Guangdong Laboratory, Chaozhou, 521011, China
[4] School of Food Science and Engineering, South China University of Technology, Guangzhou, Guangdong
* Corresponding author: Dr. Zhong-Sheng Tang, zstang_yj@gdou.edu.cn (Z. Tang).
** Corresponding author: Prof. Siming Zhu, (S. Zhu)
Address: 1 Luoqin, Yangjiang, China

Table S1. Box-Behnken response surface experimental factor levels.

	A	B	C
	**Solid-Liquid Ratio（mL/g）**	**Ultrasound time (min)**	**Ultrasound power (W)**
-1	25	20	60
0	30	30	90
1	35	40	120

1.1. Results of single-factor experiments on the yield of JP

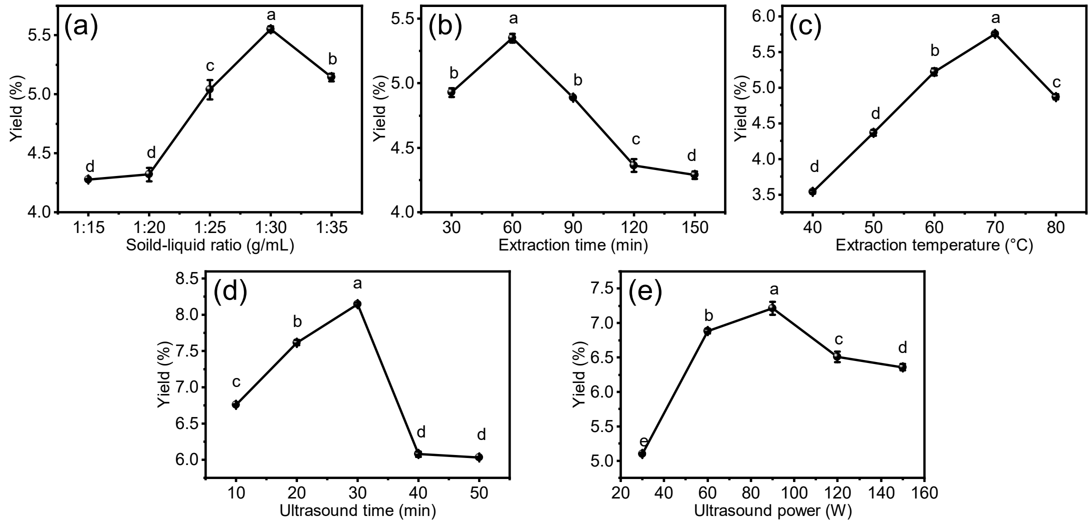

Figure S1. Influence of different factors and levels on the yield of polysaccharides. (**a**) Solid-liquid ratio, (**b**) extraction time, (**c**) extraction temperature, (**d**) ultrasonic time, (**e**) ultrasonic time. Different letters (**a, b, c, and d**) indicate significant differences（$n = 3$, $p < 0.05$）.

As shown in **Figure S1**, the influence of various factors on the yield of JP showed a similar trend. When the ratio of material to liquid increases from 1:15 to 1:30, the yield of polysaccharides increased. Further increased the ratio to 1:35, it decreases due to the excessive swelling of raw materials hindering the dissolution of polysaccharides. For other factors, excessive extraction time (ultrasonic time), high extraction temperature, and high ultrasonic power would induce the degradation of polysaccharides, thereby decreasing the yield.

1.2. Response surface and variance analysis results

1.2.1. Model Fitting and Statistical Analysis

According to the results of the single-factor experiment, the Box-Behnken of Design Expert 13 software was used to design the optimized extraction conditions of polysaccharides (**Table S2**). The quadratic regression equations between the three independent variables and the yield of polysaccharides are as follows:

Y=8.45+0.5037A-0.3120B+0.2380C-0.6073AB-0.1447AC+0.4668BC-2.23A2-1.04B2-0.8499C

Table S2. Response surface test results.

	A	B	D	
	**Solid-Liquid Ratio**	**Ultrasound time**	**Ultrasound power**	**Polysaccharide yield（%）**
1	-1	0	1	5.153±0.080
2	0	0	0	8.514±0.323

3	1	1	0	4.520±0.124
4	0	1	-1	5.689±0.196
5	0	0	0	8.395±0.316
6	0	0	0	8.438±0.287
7	-1	0	-1	4.484±0.086
8	0	0	0	8.691±0.214
9	-1	1	0	4.823±0.140
10	1	0	1	5.967±0.262
11	0	0	0	8.187±0.117
12	0	-1	1	6.491±0.123
13	0	-1	-1	6.852±0.158
14	1	0	-1	5.878±0.289
15	-1	-1	0	4.627±0.166
16	0	1	1	7.195±0.274
17	1	-1	0	6.753±0.305

The values shown in the figure are the mean ± standard deviation.

Based on the analysis of variance and the sufficiency and adaptability of the quadratic regression model (**Table S3**), the results showed that the overall regression model was significant ($p < 0.01$), and the lack-of-fit term was not significant ($F = 3.47$, $p = 0.1304 > 0.05$), indicating that the model was valid and the good fitting of the equation. The correlation coefficient R^2 of the equation was 0.9870, and the adjusted coefficient $RAdj^2$ was 0.9704. Moreover, $RAdj^2$-$RPred^2$ = 0.1209 < 0.2, which indicated the high fitting and prediction accuracy of model. The order of influence of the three parameters on the polysaccharide yield was: solid-liquid ratio > ultrasonic time > ultrasonic power.

Table S3. Variance analysis of the model.

Sources of Variance	sum of Squares	df	Mean square	F Value	P Value	Significance
Regression Model	36.71	9	4.08	59.20	<0.0001	**
A	2.03	1	2.03	29.47	0.0010	**
B	0.7788	1	0.7788	11.3	0.0120	*
C	0.4532	1	0.4532	6.58	0.0373	*
AB	1.48	1	1.48	21.41	0.0024	**
AC	0.0838	1	0.0838	1.22	0.3065	ns
BC	0.8714	1	0.8714	12.65	0.0093	**
A^2	20.86	1	20.86	302.82	<0.0001	**
B^2	4.55	1	4.55	66.03	<0.0001	**
C^2	3.04	1	3.04	44.15	<0.0001	**
Residual	0.4822	7	0.0689	--	--	--
Lack-of-Fit Term	0.3483	3	0.1161	3.47	0.1304	ns
Pure Error	0.1339	4	0.0335	--	--	--
Total	37.19	16		--	--	--

** indicates extremely significant difference ($p < 0.01$), * indicates significant difference ($p < 0.05$), and ns indicates no significant difference ($p > 0.05$). Note:R^2=0.9870，$RAdj^2$=0.9704，$RPred^2$=0.8445.

1.3. Analysis of monosaccharide composition

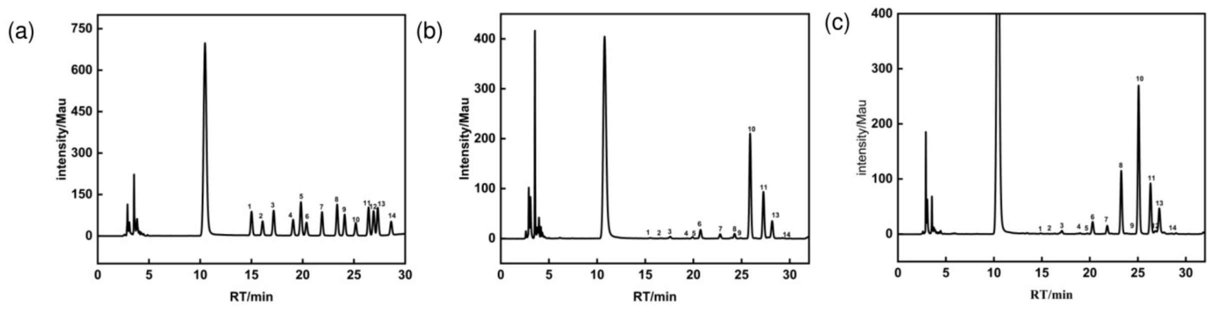

Figure. **S2** Monosaccharide composition chromatograms of jackfruit polysaccharides(JP);(a) Chromatogram of a standard mixture of 14 monosaccharides; (b) Acid hydrolysate of polysaccharides obtained by hot water extraction (HAE-JP); (c) Acid hydrolysate of polysaccharides obtained by ultrasound-assisted extraction (UAE-JP);the numbered peaks (1-14) in the standard chromatogram (a) correspond to:1 L-glucuronic acid ;2 D-mannuronic acid ;3 D-mannose Man ;4 D-glucosamine GlcN;5 D-ribose Rib;6 L-rhamnose Rham;7 D-glucuronic acid GlcUA;8 D-galacturonic acid GalUA;9 D-glucosamine GalN ;10 D-glucose Glc;11 D-galactose Gal;12 D-xylose Xyl ;13 L-arabinose Ara ;14 L-fucose Fuc.
